# 2-Eth­oxy-4-(4-methyl­phen­yl)-6-phenyl­pyridine-3-carbonitrile

**DOI:** 10.1107/S1600536812032163

**Published:** 2012-07-18

**Authors:** Shaaban K. Mohamed, Mehmet Akkurt, Antar A. Abdelhamid, Kuldip Singh, Mahoud A. A. El-Remaily

**Affiliations:** a Chemistry and Environmental Division, Manchester Metropolitan University, Manchester M1 5GD, England; b Department of Physics, Faculty of Sciences, Erciyes University, 38039 Kayseri, Turkey; c Department of Chemistry, University of Leicester, Leicester, England; d Department of Chemistry, Faculty of Science, Sohag University, 82524 Sohag, Egypt

## Abstract

The title compound, C_21_H_18_N_2_O, crystallized with two independent mol­ecules (*A* and *B*) in the asymmetric unit. In mol­ecule *A*, the central pyridine ring forms dihedral angles of 14.55 (13) and 39.14 (12)° with the terminal phenyl and benzene rings, respectively. The latter rings make a dihedral angle of 33.06 (13)° with each other. The corresponding values for mol­ecule *B* are 26.86 (13), 41.82 (12) and 38.99 (13)°, respectively. In the crystal, the *B* mol­ecules are linked *via* a pair of weak C—H⋯N hydrogen bonds, forming inversion dimers. In addition, C—H⋯π inter­actions and π–π [centroid–centroid distances = 3.5056 (16) and 3.8569 (17) Å] stacking inter­actions are observed.

## Related literature

For the bioactivity of pyridine compounds, see: Cook *et al.* (2004 ▶); Upton *et al.* (2000 ▶); Ellefson *et al.* (1978 ▶). For the synthesis of bioactive mol­ecules, see: El-Sawy *et al.* (2012 ▶); Soliman *et al.* (2012 ▶). For a similar structure, see: Patel *et al.* (2002 ▶).
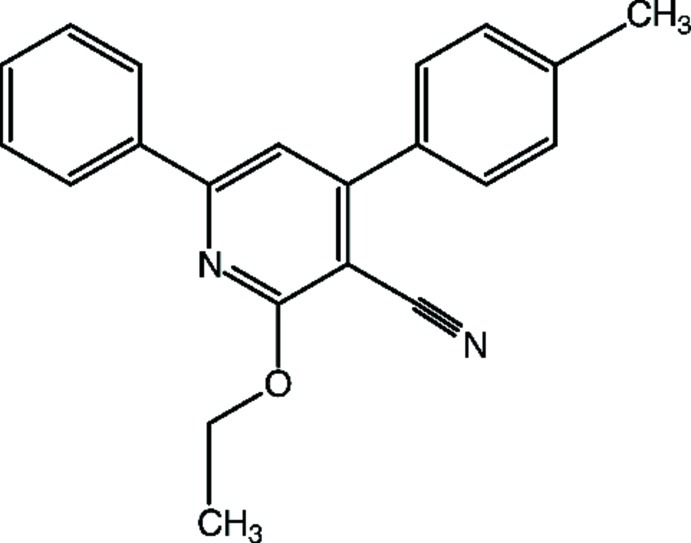


## Experimental

### 

#### Crystal data

C_21_H_18_N_2_O
*M* *_r_* = 314.37
Monoclinic, 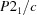

*a* = 14.786 (3) Å
*b* = 14.634 (3) Å
*c* = 15.399 (3) Å
β = 92.288 (4)°
*V* = 3329.4 (12) Å^3^
*Z* = 8
Mo *K*α radiation
μ = 0.08 mm^−1^
*T* = 150 K
0.35 × 0.15 × 0.11 mm

#### Data collection

Bruker APEX 2000 CCD area-detector diffractometer
Absorption correction: multi-scan (*SADABS*; Bruker, 2005 ▶) *T*_min_ = 0.986, *T*_max_ = 0.991
25600 measured reflections
6846 independent reflections
3334 reflections with *I* > 2σ(*I*)
*R*_int_ = 0.105

#### Refinement

*R*[*F* ^2^ > 2σ(*F* ^2^)] = 0.061
*wR*(*F* ^2^) = 0.157
*S* = 0.81
6846 reflections
433 parameters
H-atom parameters constrained
Δρ_max_ = 0.26 e Å^−3^
Δρ_min_ = −0.27 e Å^−3^

### 

Data collection: *SMART* (Bruker, 2005 ▶); cell refinement: *SAINT* (Bruker, 2005 ▶); data reduction: *SAINT*; program(s) used to solve structure: *SIR97* (Altomare *et al.*, 1999 ▶); program(s) used to refine structure: *SHELXL97* (Sheldrick, 2008 ▶); molecular graphics: *ORTEP-3 for Windows* (Farrugia, 1997 ▶); software used to prepare material for publication: *WinGX* (Farrugia, 1999 ▶) and *PLATON* (Spek, 2009 ▶).

## Supplementary Material

Crystal structure: contains datablock(s) global, I. DOI: 10.1107/S1600536812032163/su2474sup1.cif

Structure factors: contains datablock(s) I. DOI: 10.1107/S1600536812032163/su2474Isup2.hkl

Supplementary material file. DOI: 10.1107/S1600536812032163/su2474Isup3.cml

Additional supplementary materials:  crystallographic information; 3D view; checkCIF report

## Figures and Tables

**Table 1:** Hydrogen-bond geometry (Å, °) *Cg*2, *Cg*5 and *Cg*6 are the centroids of the C6–C11, C6*A*–C11*A* and C12*A*–C17*A* rings, respectively.

*D*—H⋯*A*	*D*—H	H⋯*A*	*D*⋯*A*	*D*—H⋯*A*
C13*A*—H13*A*⋯N2*A* ^i^	0.93	2.59	3.353 (3)	139
C19—H19*A*⋯*Cg*6^ii^	0.97	2.72	3.622 (3)	155
C20—H20*B*⋯*Cg*5	0.96	2.76	3.693 (3)	163
C20*A*—H20*E*⋯*Cg*2	0.96	2.83	3.746 (3)	159

Symmetry codes: (i) 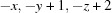; (ii) 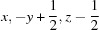.

**Fig. 1.**
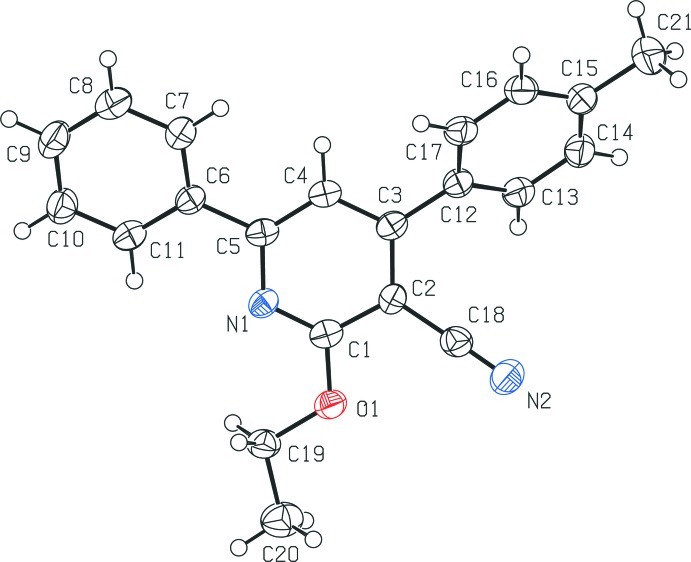
The molecular structure of molecule A in the asymmetric unit of the title compound, with the atom numbering. Displacement ellipsoids are drawn at the 50% probability level.

**Fig. 2.**
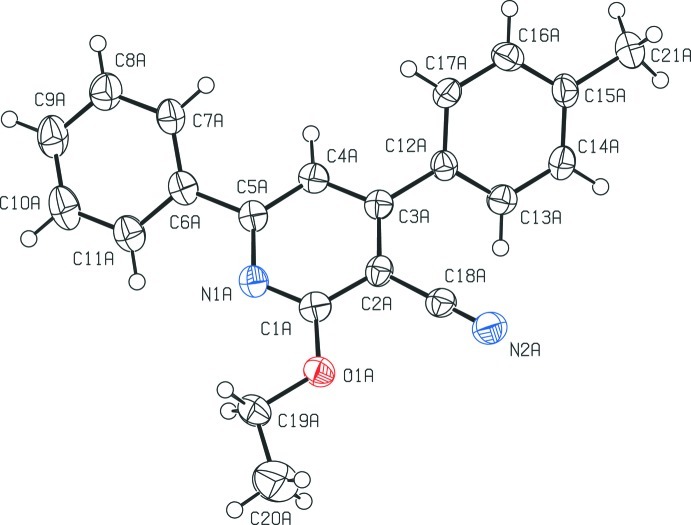
The molecular structure of molecule B in the asymmetric unit of the title compound, with the atom numbering. Displacement ellipsoids are drawn at the 50% probability level.

**Fig. 3.**
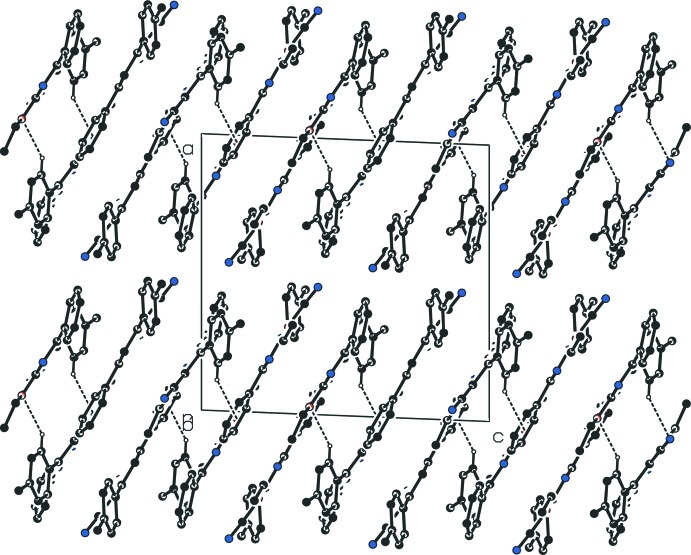
A view along the b-axis of the crystal packing of the title compound. The C-H···N interactions are shown as dashed lines [the H atoms not involved in hydrogen bonding have been omitted for clarity].

## supplementary crystallographic information

### Comment

Pyridine containing compounds possess a wide range of biological properties (Cook *et al.*, 2004; Upton *et al.*, 2000; Ellefson *et al.*, 1978). Functionalized pyridine derivatives can act as antifungal (Cook *et al.*, 2004), antifertility (Upton *et al.*, 2000) and antiarrhythmic agents (Ellefson *et al.*, 1978). We herein report on the synthesis and crystal structure of the title compound as a part of our on-going project on the synthesis of bioactive molecules (El-Sawy *et al.*, 2012; Soliman *et al.*, 2012).

The molecular structures of the two independent molecules (A and B) of the title compound have similar conformations (Figs. 1 & 2). In molecule A the N1/C1–C5 pyridine ring forms dihedral angles of 14.55 (13) and 39.14 (12)° with the C6–C11 phenyl and C12–C17 benzene rings, respectively. The latter rings make a dihedral angle of 33.06 (13)° with each other. The corresponding values for the B molecule are 26.86 (13), 41.82 (12) and 38.99 (13)°, respectively. The values of the bond lengths and bond angles are in the normal range and are comparable to those reported for a similar structure (Patel *et al.*, 2002).

In the crystal, the B molecules are linked via pairs of C—H···N hydrogen bonds to form inversion dimers that stack together with the A molecules along the c axis direction (Table 1 and Fig. 3). Inter- and intra-molecular C—H···π interactions (Table 1) and π-π [*Cg*1···*Cg*4(*x*, *y*, *z*) = 3.5056 (16) Å and *Cg*2···*Cg*4(*x*, 1/2 - *y*, -1/2 + *z*) = 3.8569 (17) Å; where *Cg*1, *Cg*2 and *Cg*4 are the centroids of the N1/C1–C5, C6–C11 and N1A/C1A–C5A rings, respectively] interactions contribute to the stabilization of the crystal packing.

### Experimental

The title compound was prepared by heating a mixture of (2*E*)-3-(4-methylphenyl)-1-phenylprop-2-en-1-one (222 mg, 1 mmol), propanedinitrile (66 mg, 1 mmol) and sodium methoxide (10 mg) as a catalyst in 50 ml e thanol at 350 K for 7 h. The solid product that resulted on cooling was filtered off, dried and recrystallized from acetone. Single crystals suitable for X-ray analyses were grown by slow evaporation of an acetone solution of the title compound over 24 h [*M*.p. 383 K].

### Refinement

All H atoms were positioned geometrically with C—H = 0.93 Å (aromatic), 0.97 Å (methylene) and 0.96 Å (methyl). The *U*_iso_ values were constrained to be 1.5U_eq_ of the carrier atom for methyl H atoms and 1.2*U*_eq_ for the remaining H atoms. Owing to poor agreement the reflection (1 0 0, -1 0 2, -2 1 1, 3 3 1, 5 1 6) were omitted from the final cycles of refinement.

### Crystal data

**Table:**

C_21_H_18_N_2_O	*F*(000) = 1328
*M**_r_* = 314.37	*D*_x_ = 1.254 Mg m^−^^3^
Monoclinic, *P*2_1_/*c*	Mo *K*α radiation, λ = 0.71073 Å
Hall symbol: -P 2ybc	Cell parameters from 744 reflections
*a* = 14.786 (3) Å	θ = 2.3–28.2°
*b* = 14.634 (3) Å	µ = 0.08 mm^−^^1^
*c* = 15.399 (3) Å	*T* = 150 K
β = 92.288 (4)°	Block, colourless
*V* = 3329.4 (12) Å^3^	0.35 × 0.15 × 0.11 mm
*Z* = 8	

### Data collection

**Table:**

Bruker APEX 2000 CCD area-detector diffractometer	6846 independent reflections
Radiation source: fine-focus sealed tube	3334 reflections with *I* > 2σ(*I*)
Graphite monochromator	*R*_int_ = 0.105
phi and ω scans	θ_max_ = 26.5°, θ_min_ = 1.9°
Absorption correction: multi-scan (*SADABS*; Bruker, 2005)	*h* = −18→18
*T*_min_ = 0.986, *T*_max_ = 0.991	*k* = −18→18
25600 measured reflections	*l* = −19→19

### Refinement

**Table:**

Refinement on *F*^2^	Primary atom site location: structure-invariant direct methods
Least-squares matrix: full	Secondary atom site location: difference Fourier map
*R*[*F*^2^ > 2σ(*F*^2^)] = 0.061	Hydrogen site location: inferred from neighbouring sites
*wR*(*F*^2^) = 0.157	H-atom parameters constrained
*S* = 0.81	*w* = 1/[σ^2^(*F*_o_^2^) + (0.0671*P*)^2^] where *P* = (*F*_o_^2^ + 2*F*_c_^2^)/3
6846 reflections	(Δ/σ)_max_ < 0.001
433 parameters	Δρ_max_ = 0.26 e Å^−^^3^
0 restraints	Δρ_min_ = −0.27 e Å^−^^3^

### Special details

**Table:**

Geometry. Bond distances, angles *etc*. have been calculated using the rounded fractional coordinates. All su's are estimated from the variances of the (full) variance-covariance matrix. The cell e.s.d.'s are taken into account in the estimation of distances, angles and torsion angles
Refinement. Refinement on *F*^2^ for ALL reflections except those flagged by the user for potential systematic errors. Weighted *R*-factors *wR* and all goodnesses of fit *S* are based on *F*^2^, conventional *R*-factors *R* are based on *F*, with *F* set to zero for negative *F*^2^. The observed criterion of *F*^2^ > σ(*F*^2^) is used only for calculating -*R*-factor-obs *etc*. and is not relevant to the choice of reflections for refinement. *R*-factors based on *F*^2^ are statistically about twice as large as those based on *F*, and *R*-factors based on ALL data will be even larger.

### Fractional atomic coordinates and isotropic or equivalent isotropic displacement parameters (Å^2^)

**Table:**

	*x*	*y*	*z*	*U*_iso_*/*U*_eq_	
O1	0.31241 (11)	0.13660 (11)	0.80183 (11)	0.0368 (7)	
N1	0.19322 (14)	0.21501 (13)	0.73551 (13)	0.0310 (8)	
N2	0.46150 (16)	0.27830 (16)	0.90471 (17)	0.0496 (10)	
C1	0.27212 (17)	0.21613 (17)	0.77761 (17)	0.0309 (9)	
C2	0.31949 (16)	0.29620 (17)	0.80252 (17)	0.0301 (9)	
C3	0.28257 (17)	0.38042 (17)	0.77720 (16)	0.0304 (9)	
C4	0.19991 (17)	0.37776 (17)	0.73149 (17)	0.0315 (9)	
C5	0.15556 (17)	0.29653 (17)	0.71266 (16)	0.0298 (9)	
C6	0.06623 (17)	0.29272 (17)	0.66582 (16)	0.0309 (9)	
C7	0.01119 (18)	0.36936 (19)	0.65580 (18)	0.0420 (11)	
C8	−0.0717 (2)	0.3645 (2)	0.6117 (2)	0.0488 (11)	
C9	−0.10134 (19)	0.2828 (2)	0.57656 (19)	0.0461 (11)	
C10	−0.04868 (19)	0.2054 (2)	0.58673 (19)	0.0427 (11)	
C11	0.03373 (17)	0.21074 (18)	0.63119 (17)	0.0356 (10)	
C12	0.32673 (18)	0.46887 (17)	0.79885 (17)	0.0335 (9)	
C13	0.41954 (18)	0.48137 (18)	0.79421 (18)	0.0390 (10)	
C14	0.4591 (2)	0.56581 (19)	0.81318 (19)	0.0452 (11)	
C15	0.4068 (2)	0.63955 (19)	0.83808 (18)	0.0433 (11)	
C16	0.3143 (2)	0.62681 (18)	0.84187 (18)	0.0415 (11)	
C17	0.27505 (19)	0.54326 (17)	0.82279 (17)	0.0375 (10)	
C18	0.39916 (19)	0.28677 (17)	0.85804 (19)	0.0364 (10)	
C19	0.26215 (18)	0.05307 (16)	0.78455 (19)	0.0386 (10)	
C20	0.3204 (2)	−0.02487 (19)	0.8163 (2)	0.0537 (11)	
C21	0.4503 (2)	0.72999 (19)	0.8612 (2)	0.0616 (12)	
O1A	0.02702 (12)	0.23795 (11)	0.87536 (12)	0.0366 (7)	
N1A	0.15020 (15)	0.17393 (14)	0.94927 (14)	0.0327 (8)	
N2A	0.03715 (15)	0.46836 (16)	0.87169 (16)	0.0444 (9)	
C1A	0.10422 (18)	0.24720 (18)	0.92368 (17)	0.0329 (9)	
C2A	0.13030 (17)	0.33768 (17)	0.94292 (17)	0.0309 (9)	
C3A	0.20771 (17)	0.35143 (17)	0.99684 (17)	0.0295 (9)	
C4A	0.25586 (18)	0.27376 (16)	1.02280 (17)	0.0321 (9)	
C5A	0.22706 (17)	0.18773 (17)	0.99754 (17)	0.0305 (9)	
C6A	0.27821 (18)	0.10427 (17)	1.02506 (16)	0.0329 (9)	
C7A	0.37064 (19)	0.10723 (18)	1.04322 (17)	0.0378 (10)	
C8A	0.4164 (2)	0.02925 (19)	1.06989 (19)	0.0458 (11)	
C9A	0.3721 (2)	−0.05204 (19)	1.07808 (19)	0.0499 (11)	
C10A	0.2803 (2)	−0.05577 (19)	1.05882 (19)	0.0525 (11)	
C11A	0.2333 (2)	0.02161 (18)	1.03310 (18)	0.0417 (10)	
C12A	0.23582 (17)	0.44329 (16)	1.02802 (16)	0.0304 (9)	
C13A	0.17244 (17)	0.50456 (17)	1.05695 (17)	0.0332 (9)	
C14A	0.19849 (18)	0.58882 (17)	1.09059 (18)	0.0374 (10)	
C15A	0.28916 (19)	0.61318 (17)	1.09688 (18)	0.0378 (10)	
C16A	0.35246 (18)	0.55226 (17)	1.06731 (18)	0.0377 (10)	
C17A	0.32693 (17)	0.46770 (17)	1.03462 (17)	0.0329 (9)	
C18A	0.07827 (17)	0.41064 (19)	0.90492 (18)	0.0338 (10)	
C19A	−0.00194 (19)	0.14604 (18)	0.85355 (19)	0.0432 (11)	
C20A	−0.0929 (2)	0.1560 (2)	0.8145 (2)	0.0779 (17)	
C21A	0.3180 (2)	0.70398 (18)	1.1353 (2)	0.0578 (13)	
H4	0.17340	0.43240	0.71290	0.0380*	
H7	0.03060	0.42490	0.67920	0.0500*	
H8	−0.10760	0.41640	0.60570	0.0580*	
H9	−0.15680	0.27980	0.54600	0.0550*	
H10	−0.06880	0.15000	0.56370	0.0510*	
H11	0.06860	0.15820	0.63830	0.0430*	
H13	0.45580	0.43270	0.77820	0.0470*	
H14	0.52130	0.57290	0.80910	0.0540*	
H16	0.27790	0.67550	0.85760	0.0500*	
H17	0.21270	0.53660	0.82600	0.0450*	
H19A	0.24820	0.04690	0.72270	0.0460*	
H19B	0.20580	0.05380	0.81470	0.0460*	
H20A	0.28840	−0.08140	0.80700	0.0810*	
H20B	0.33480	−0.01730	0.87720	0.0810*	
H20C	0.37530	−0.02580	0.78500	0.0810*	
H21A	0.50470	0.73700	0.82980	0.0920*	
H21B	0.46490	0.73180	0.92250	0.0920*	
H21C	0.40910	0.77870	0.84600	0.0920*	
H4A	0.30830	0.27990	1.05770	0.0390*	
H7A	0.40200	0.16190	1.03740	0.0450*	
H8A	0.47840	0.03210	1.08250	0.0550*	
H9A	0.40350	−0.10410	1.09640	0.0600*	
H10A	0.24980	−0.11100	1.06320	0.0630*	
H11A	0.17130	0.01840	1.02110	0.0500*	
H13A	0.11140	0.48900	1.05380	0.0400*	
H14A	0.15480	0.62940	1.10910	0.0450*	
H16A	0.41330	0.56850	1.06950	0.0450*	
H17A	0.37080	0.42690	1.01690	0.0390*	
H19C	−0.00280	0.10820	0.90520	0.0520*	
H19D	0.03830	0.11850	0.81270	0.0520*	
H20D	−0.11590	0.09710	0.79740	0.1170*	
H20E	−0.09090	0.19490	0.76440	0.1170*	
H20F	−0.13180	0.18270	0.85610	0.1170*	
H21D	0.26760	0.73170	1.16270	0.0870*	
H21E	0.33810	0.74330	1.09010	0.0870*	
H21F	0.36650	0.69470	1.17770	0.0870*	

### Atomic displacement parameters (Å^2^)

**Table:**

	*U*^11^	*U*^22^	*U*^33^	*U*^12^	*U*^13^	*U*^23^
O1	0.0394 (11)	0.0332 (11)	0.0373 (12)	0.0053 (9)	−0.0054 (9)	0.0001 (8)
N1	0.0321 (13)	0.0354 (13)	0.0254 (13)	0.0033 (10)	−0.0015 (10)	−0.0003 (10)
N2	0.0409 (15)	0.0571 (17)	0.0500 (18)	−0.0037 (12)	−0.0075 (13)	0.0124 (13)
C1	0.0334 (16)	0.0345 (16)	0.0252 (16)	0.0067 (12)	0.0051 (12)	0.0017 (12)
C2	0.0264 (14)	0.0369 (16)	0.0269 (16)	−0.0011 (12)	0.0002 (12)	0.0013 (12)
C3	0.0329 (15)	0.0363 (16)	0.0222 (15)	0.0027 (12)	0.0026 (12)	0.0025 (12)
C4	0.0339 (16)	0.0313 (15)	0.0294 (16)	0.0061 (12)	0.0020 (13)	0.0034 (12)
C5	0.0374 (16)	0.0326 (15)	0.0196 (15)	0.0063 (12)	0.0039 (12)	0.0008 (11)
C6	0.0342 (16)	0.0355 (16)	0.0228 (16)	0.0016 (12)	−0.0010 (12)	0.0010 (12)
C7	0.0391 (18)	0.0401 (17)	0.046 (2)	0.0010 (13)	−0.0098 (15)	−0.0003 (14)
C8	0.0462 (19)	0.0463 (19)	0.053 (2)	0.0087 (15)	−0.0093 (16)	0.0054 (15)
C9	0.0384 (18)	0.062 (2)	0.0372 (19)	−0.0029 (16)	−0.0088 (14)	0.0052 (15)
C10	0.0412 (18)	0.0483 (18)	0.0381 (19)	−0.0033 (14)	−0.0058 (15)	−0.0012 (14)
C11	0.0370 (17)	0.0394 (17)	0.0302 (17)	0.0060 (13)	−0.0003 (13)	0.0023 (13)
C12	0.0383 (16)	0.0363 (16)	0.0255 (16)	−0.0028 (13)	−0.0035 (13)	0.0049 (12)
C13	0.0372 (17)	0.0390 (17)	0.0403 (19)	−0.0002 (13)	−0.0030 (14)	0.0078 (13)
C14	0.0392 (17)	0.0496 (19)	0.046 (2)	−0.0108 (15)	−0.0081 (14)	0.0126 (15)
C15	0.051 (2)	0.0393 (18)	0.0384 (19)	−0.0075 (15)	−0.0141 (15)	0.0070 (14)
C16	0.051 (2)	0.0323 (16)	0.0405 (19)	0.0028 (14)	−0.0076 (15)	0.0011 (13)
C17	0.0413 (17)	0.0395 (17)	0.0314 (17)	−0.0004 (13)	−0.0006 (13)	0.0091 (13)
C18	0.0347 (17)	0.0359 (17)	0.0387 (19)	−0.0013 (13)	0.0045 (14)	0.0056 (13)
C19	0.0473 (17)	0.0295 (15)	0.0386 (18)	0.0046 (13)	−0.0035 (14)	−0.0009 (13)
C20	0.066 (2)	0.0481 (19)	0.047 (2)	0.0114 (16)	0.0008 (17)	−0.0010 (15)
C21	0.072 (2)	0.047 (2)	0.064 (2)	−0.0133 (17)	−0.0192 (19)	0.0006 (17)
O1A	0.0376 (11)	0.0363 (11)	0.0353 (12)	−0.0025 (8)	−0.0041 (9)	−0.0048 (8)
N1A	0.0391 (14)	0.0331 (13)	0.0259 (13)	0.0002 (10)	0.0015 (11)	−0.0056 (10)
N2A	0.0389 (15)	0.0475 (16)	0.0465 (17)	0.0019 (12)	−0.0010 (12)	−0.0030 (12)
C1A	0.0343 (16)	0.0403 (17)	0.0242 (16)	0.0007 (13)	0.0040 (13)	−0.0031 (13)
C2A	0.0334 (16)	0.0305 (15)	0.0293 (16)	0.0041 (12)	0.0069 (13)	−0.0001 (12)
C3A	0.0311 (15)	0.0321 (15)	0.0257 (15)	0.0012 (12)	0.0070 (12)	−0.0005 (12)
C4A	0.0349 (16)	0.0356 (16)	0.0260 (16)	−0.0006 (12)	0.0025 (12)	−0.0040 (12)
C5A	0.0367 (16)	0.0318 (15)	0.0232 (16)	0.0004 (12)	0.0053 (12)	−0.0011 (11)
C6A	0.0459 (18)	0.0327 (15)	0.0201 (15)	−0.0020 (13)	0.0021 (13)	−0.0029 (12)
C7A	0.0482 (18)	0.0303 (16)	0.0349 (18)	0.0014 (13)	0.0003 (14)	−0.0025 (12)
C8A	0.055 (2)	0.0416 (18)	0.040 (2)	0.0058 (15)	−0.0081 (15)	−0.0004 (14)
C9A	0.073 (2)	0.0354 (18)	0.040 (2)	0.0053 (16)	−0.0133 (17)	0.0054 (14)
C10A	0.080 (2)	0.0333 (18)	0.044 (2)	−0.0045 (16)	−0.0015 (18)	0.0032 (14)
C11A	0.0537 (19)	0.0367 (17)	0.0345 (18)	−0.0027 (14)	−0.0022 (14)	−0.0004 (13)
C12A	0.0357 (16)	0.0273 (14)	0.0279 (16)	0.0005 (12)	−0.0009 (12)	−0.0009 (12)
C13A	0.0339 (15)	0.0343 (15)	0.0311 (17)	0.0002 (12)	−0.0013 (12)	0.0047 (12)
C14A	0.0424 (17)	0.0292 (15)	0.0406 (19)	0.0069 (13)	0.0000 (14)	0.0021 (13)
C15A	0.0464 (18)	0.0254 (15)	0.0409 (19)	0.0016 (13)	−0.0080 (14)	0.0017 (12)
C16A	0.0347 (16)	0.0364 (16)	0.0414 (19)	−0.0064 (13)	−0.0054 (13)	0.0077 (13)
C17A	0.0318 (16)	0.0328 (15)	0.0339 (17)	0.0051 (12)	−0.0010 (12)	0.0012 (12)
C18A	0.0307 (16)	0.0369 (17)	0.0339 (18)	0.0004 (13)	0.0036 (13)	−0.0049 (13)
C19A	0.0432 (18)	0.0399 (18)	0.046 (2)	−0.0060 (14)	−0.0060 (15)	−0.0070 (14)
C20A	0.070 (3)	0.079 (3)	0.084 (3)	−0.009 (2)	−0.007 (2)	−0.010 (2)
C21A	0.055 (2)	0.0313 (17)	0.086 (3)	−0.0007 (15)	−0.0104 (18)	−0.0043 (16)

### Geometric parameters (Å, º)

**Table:**

O1—C1	1.353 (3)	C20—H20B	0.9600
O1—C19	1.450 (3)	C20—H20C	0.9600
O1A—C1A	1.344 (3)	C21—H21B	0.9600
O1A—C19A	1.447 (3)	C21—H21C	0.9600
N1—C1	1.312 (3)	C21—H21A	0.9600
N1—C5	1.357 (3)	C1A—C2A	1.407 (4)
N2—C18	1.153 (4)	C2A—C3A	1.402 (4)
N1A—C1A	1.321 (3)	C2A—C18A	1.428 (4)
N1A—C5A	1.348 (3)	C3A—C4A	1.391 (4)
N2A—C18A	1.149 (4)	C3A—C12A	1.481 (3)
C1—C2	1.410 (4)	C4A—C5A	1.380 (3)
C2—C18	1.434 (4)	C5A—C6A	1.489 (4)
C2—C3	1.397 (4)	C6A—C7A	1.385 (4)
C3—C4	1.386 (4)	C6A—C11A	1.388 (4)
C3—C12	1.482 (4)	C7A—C8A	1.381 (4)
C4—C5	1.383 (4)	C8A—C9A	1.366 (4)
C5—C6	1.481 (4)	C9A—C10A	1.379 (4)
C6—C7	1.391 (4)	C10A—C11A	1.378 (4)
C6—C11	1.391 (4)	C12A—C13A	1.384 (4)
C7—C8	1.379 (4)	C12A—C17A	1.394 (4)
C8—C9	1.377 (4)	C13A—C14A	1.386 (4)
C9—C10	1.380 (4)	C14A—C15A	1.387 (4)
C10—C11	1.376 (4)	C15A—C16A	1.383 (4)
C12—C13	1.389 (4)	C15A—C21A	1.509 (4)
C12—C17	1.388 (4)	C16A—C17A	1.383 (4)
C13—C14	1.393 (4)	C19A—C20A	1.459 (4)
C14—C15	1.390 (4)	C4A—H4A	0.9300
C15—C21	1.508 (4)	C7A—H7A	0.9300
C15—C16	1.384 (4)	C8A—H8A	0.9300
C16—C17	1.380 (4)	C9A—H9A	0.9300
C19—C20	1.499 (4)	C10A—H10A	0.9300
C4—H4	0.9300	C11A—H11A	0.9300
C7—H7	0.9300	C13A—H13A	0.9300
C8—H8	0.9300	C14A—H14A	0.9300
C9—H9	0.9300	C16A—H16A	0.9300
C10—H10	0.9300	C17A—H17A	0.9300
C11—H11	0.9300	C19A—H19C	0.9700
C13—H13	0.9300	C19A—H19D	0.9700
C14—H14	0.9300	C20A—H20D	0.9600
C16—H16	0.9300	C20A—H20E	0.9600
C17—H17	0.9300	C20A—H20F	0.9600
C19—H19A	0.9700	C21A—H21D	0.9600
C19—H19B	0.9700	C21A—H21E	0.9600
C20—H20A	0.9600	C21A—H21F	0.9600
			
C1—O1—C19	117.25 (19)	H21A—C21—H21B	109.00
C1A—O1A—C19A	117.3 (2)	C15—C21—H21A	109.00
C1—N1—C5	117.7 (2)	O1A—C1A—N1A	119.9 (2)
C1A—N1A—C5A	117.1 (2)	O1A—C1A—C2A	115.5 (2)
N1—C1—C2	124.5 (2)	N1A—C1A—C2A	124.6 (2)
O1—C1—N1	119.9 (2)	C1A—C2A—C3A	118.1 (2)
O1—C1—C2	115.6 (2)	C1A—C2A—C18A	118.6 (2)
C1—C2—C3	118.2 (2)	C3A—C2A—C18A	123.3 (2)
C1—C2—C18	118.0 (2)	C2A—C3A—C4A	116.7 (2)
C3—C2—C18	123.6 (2)	C2A—C3A—C12A	122.3 (2)
C2—C3—C4	116.3 (2)	C4A—C3A—C12A	121.0 (2)
C4—C3—C12	120.7 (2)	C3A—C4A—C5A	121.0 (2)
C2—C3—C12	123.0 (2)	N1A—C5A—C4A	122.4 (2)
C3—C4—C5	122.1 (2)	N1A—C5A—C6A	116.2 (2)
C4—C5—C6	122.8 (2)	C4A—C5A—C6A	121.4 (2)
N1—C5—C6	116.2 (2)	C5A—C6A—C7A	121.2 (2)
N1—C5—C4	121.0 (2)	C5A—C6A—C11A	120.1 (2)
C5—C6—C7	122.0 (2)	C7A—C6A—C11A	118.8 (2)
C5—C6—C11	120.5 (2)	C6A—C7A—C8A	120.2 (2)
C7—C6—C11	117.5 (2)	C7A—C8A—C9A	121.1 (3)
C6—C7—C8	121.2 (3)	C8A—C9A—C10A	119.1 (3)
C7—C8—C9	120.1 (3)	C9A—C10A—C11A	120.7 (3)
C8—C9—C10	119.9 (3)	C6A—C11A—C10A	120.3 (3)
C9—C10—C11	119.6 (3)	C3A—C12A—C13A	120.5 (2)
C6—C11—C10	121.7 (2)	C3A—C12A—C17A	120.9 (2)
C13—C12—C17	117.8 (2)	C13A—C12A—C17A	118.4 (2)
C3—C12—C13	122.1 (2)	C12A—C13A—C14A	121.0 (2)
C3—C12—C17	120.2 (2)	C13A—C14A—C15A	120.5 (2)
C12—C13—C14	120.9 (2)	C14A—C15A—C16A	118.5 (2)
C13—C14—C15	120.9 (3)	C14A—C15A—C21A	120.7 (2)
C14—C15—C21	120.6 (3)	C16A—C15A—C21A	120.8 (2)
C16—C15—C21	121.4 (3)	C15A—C16A—C17A	121.2 (2)
C14—C15—C16	117.9 (3)	C12A—C17A—C16A	120.3 (2)
C15—C16—C17	121.2 (3)	N2A—C18A—C2A	177.8 (3)
C12—C17—C16	121.3 (3)	O1A—C19A—C20A	105.2 (2)
N2—C18—C2	177.9 (3)	C3A—C4A—H4A	119.00
O1—C19—C20	107.3 (2)	C5A—C4A—H4A	119.00
C5—C4—H4	119.00	C6A—C7A—H7A	120.00
C3—C4—H4	119.00	C8A—C7A—H7A	120.00
C6—C7—H7	119.00	C7A—C8A—H8A	119.00
C8—C7—H7	119.00	C9A—C8A—H8A	119.00
C9—C8—H8	120.00	C8A—C9A—H9A	120.00
C7—C8—H8	120.00	C10A—C9A—H9A	121.00
C10—C9—H9	120.00	C9A—C10A—H10A	120.00
C8—C9—H9	120.00	C11A—C10A—H10A	120.00
C9—C10—H10	120.00	C6A—C11A—H11A	120.00
C11—C10—H10	120.00	C10A—C11A—H11A	120.00
C10—C11—H11	119.00	C12A—C13A—H13A	120.00
C6—C11—H11	119.00	C14A—C13A—H13A	119.00
C12—C13—H13	120.00	C13A—C14A—H14A	120.00
C14—C13—H13	120.00	C15A—C14A—H14A	120.00
C13—C14—H14	120.00	C15A—C16A—H16A	119.00
C15—C14—H14	120.00	C17A—C16A—H16A	119.00
C17—C16—H16	119.00	C12A—C17A—H17A	120.00
C15—C16—H16	119.00	C16A—C17A—H17A	120.00
C12—C17—H17	119.00	O1A—C19A—H19C	111.00
C16—C17—H17	119.00	O1A—C19A—H19D	111.00
O1—C19—H19B	110.00	C20A—C19A—H19C	111.00
O1—C19—H19A	110.00	C20A—C19A—H19D	111.00
H19A—C19—H19B	109.00	H19C—C19A—H19D	109.00
C20—C19—H19B	110.00	C19A—C20A—H20D	110.00
C20—C19—H19A	110.00	C19A—C20A—H20E	109.00
C19—C20—H20B	109.00	C19A—C20A—H20F	109.00
C19—C20—H20A	109.00	H20D—C20A—H20E	110.00
H20A—C20—H20C	110.00	H20D—C20A—H20F	109.00
C19—C20—H20C	109.00	H20E—C20A—H20F	109.00
H20A—C20—H20B	109.00	C15A—C21A—H21D	109.00
H20B—C20—H20C	109.00	C15A—C21A—H21E	110.00
C15—C21—H21B	109.00	C15A—C21A—H21F	109.00
C15—C21—H21C	109.00	H21D—C21A—H21E	109.00
H21A—C21—H21C	110.00	H21D—C21A—H21F	109.00
H21B—C21—H21C	110.00	H21E—C21A—H21F	109.00
			
C19—O1—C1—N1	−4.1 (3)	C3—C12—C13—C14	178.7 (3)
C19—O1—C1—C2	174.5 (2)	C12—C13—C14—C15	0.6 (4)
C1—O1—C19—C20	179.6 (2)	C13—C14—C15—C16	−1.1 (4)
C19A—O1A—C1A—C2A	179.6 (2)	C13—C14—C15—C21	178.1 (3)
C1A—O1A—C19A—C20A	170.9 (2)	C21—C15—C16—C17	−178.3 (3)
C19A—O1A—C1A—N1A	−0.1 (3)	C14—C15—C16—C17	0.9 (4)
C5—N1—C1—O1	180.0 (2)	C15—C16—C17—C12	−0.2 (4)
C5—N1—C1—C2	1.6 (4)	O1A—C1A—C2A—C3A	176.8 (2)
C1—N1—C5—C4	1.4 (4)	N1A—C1A—C2A—C3A	−3.6 (4)
C1—N1—C5—C6	−179.6 (2)	N1A—C1A—C2A—C18A	174.8 (3)
C5A—N1A—C1A—O1A	180.0 (2)	O1A—C1A—C2A—C18A	−4.9 (4)
C1A—N1A—C5A—C6A	−179.5 (2)	C1A—C2A—C3A—C12A	−173.9 (2)
C1A—N1A—C5A—C4A	2.5 (4)	C18A—C2A—C3A—C4A	−174.5 (2)
C5A—N1A—C1A—C2A	0.4 (4)	C1A—C2A—C3A—C4A	3.8 (4)
N1—C1—C2—C18	172.0 (2)	C18A—C2A—C3A—C12A	7.8 (4)
N1—C1—C2—C3	−3.3 (4)	C2A—C3A—C4A—C5A	−1.2 (4)
O1—C1—C2—C3	178.2 (2)	C2A—C3A—C12A—C17A	−141.8 (3)
O1—C1—C2—C18	−6.5 (4)	C4A—C3A—C12A—C13A	−134.9 (3)
C1—C2—C3—C4	2.0 (4)	C2A—C3A—C12A—C13A	42.7 (4)
C1—C2—C3—C12	−179.4 (2)	C12A—C3A—C4A—C5A	176.5 (2)
C18—C2—C3—C4	−173.0 (2)	C4A—C3A—C12A—C17A	40.6 (4)
C18—C2—C3—C12	5.6 (4)	C3A—C4A—C5A—N1A	−2.1 (4)
C4—C3—C12—C17	38.4 (4)	C3A—C4A—C5A—C6A	−180.0 (2)
C2—C3—C12—C13	41.3 (4)	N1A—C5A—C6A—C11A	−26.0 (4)
C2—C3—C12—C17	−140.2 (3)	C4A—C5A—C6A—C7A	−27.8 (4)
C12—C3—C4—C5	−177.9 (2)	C4A—C5A—C6A—C11A	152.0 (3)
C2—C3—C4—C5	0.7 (4)	N1A—C5A—C6A—C7A	154.2 (2)
C4—C3—C12—C13	−140.1 (3)	C11A—C6A—C7A—C8A	−0.8 (4)
C3—C4—C5—C6	178.5 (2)	C5A—C6A—C11A—C10A	−179.8 (3)
C3—C4—C5—N1	−2.6 (4)	C5A—C6A—C7A—C8A	179.0 (2)
N1—C5—C6—C7	165.3 (2)	C7A—C6A—C11A—C10A	0.1 (4)
C4—C5—C6—C7	−15.7 (4)	C6A—C7A—C8A—C9A	0.6 (4)
C4—C5—C6—C11	165.4 (2)	C7A—C8A—C9A—C10A	0.4 (4)
N1—C5—C6—C11	−13.6 (3)	C8A—C9A—C10A—C11A	−1.1 (4)
C11—C6—C7—C8	−1.2 (4)	C9A—C10A—C11A—C6A	0.9 (4)
C5—C6—C11—C10	−179.4 (2)	C17A—C12A—C13A—C14A	0.9 (4)
C5—C6—C7—C8	179.8 (3)	C3A—C12A—C17A—C16A	−177.2 (2)
C7—C6—C11—C10	1.6 (4)	C3A—C12A—C13A—C14A	176.5 (2)
C6—C7—C8—C9	−0.1 (4)	C13A—C12A—C17A—C16A	−1.7 (4)
C7—C8—C9—C10	1.1 (4)	C12A—C13A—C14A—C15A	−0.7 (4)
C8—C9—C10—C11	−0.7 (4)	C13A—C14A—C15A—C16A	1.2 (4)
C9—C10—C11—C6	−0.6 (4)	C13A—C14A—C15A—C21A	−178.6 (3)
C17—C12—C13—C14	0.1 (4)	C21A—C15A—C16A—C17A	177.9 (3)
C3—C12—C17—C16	−178.9 (2)	C14A—C15A—C16A—C17A	−2.0 (4)
C13—C12—C17—C16	−0.4 (4)	C15A—C16A—C17A—C12A	2.2 (4)

### Hydrogen-bond geometry (Å, º)

Cg2, Cg5 and Cg6 are the centroids of the C6–C11, C6A–C11A and C12A–C17A rings, respectively.

**Table:**

*D*—H···*A*	*D*—H	H···*A*	*D*···*A*	*D*—H···*A*
C13*A*—H13*A*···N2*A*^i^	0.93	2.59	3.353 (3)	139
C19—H19*A*···*Cg*6^ii^	0.97	2.72	3.622 (3)	155
C20—H20*B*···*Cg*5	0.96	2.76	3.693 (3)	163
C20*A*—H20*E*···*Cg*2	0.96	2.83	3.746 (3)	159

Symmetry codes: (i) −*x*, −*y*+1, −*z*+2; (ii) *x*, −*y*+1/2, *z*−1/2.
